# Improving mental health literacy in year 9 high school children across Wales: a protocol for a randomised control treatment trial (RCT) of a mental health literacy programme across an entire country

**DOI:** 10.1186/s12889-020-08736-z

**Published:** 2020-05-19

**Authors:** Nicola J. Simkiss, Nicola S. Gray, Greg Malone, Andrew Kemp, Robert J. Snowden

**Affiliations:** 1 Department of Psychology, Swansea University, Swansea, UK; 2 Caswell Clinic, Swansea Bay University Health Boar, Bridgend, UK; 3 Action for Children, Head Office: 3 The Boulevard, Ascot Road, Watford, UK; 4 School of Psychology, Cardiff University, Cardiff, Wales CF10 3AT UK

**Keywords:** Mental health literacy, Randomised control trial, Early intervention, Self-stigma, Stigma, Education, Adolescence, Help-seeking efficacy, School-based intervention

## Abstract

**Background:**

Adolescence is a crucial period for developing and maintaining good habits for mental health and well-being. This is important for future mental health, as most mental health problems manifest during adolescence. Mental health literacy is the foundation for mental health prevention, stigma reduction, and increased help-seeking efficacy particularly among adolescents. The mental health literacy programme “The Guide”, which was developed in Canada, has shown success in increasing mental health literacy in North American 16–17 year olds. “The Guide Cymru” is an adaptation of The Guide designed for a younger age group (13–14 year olds) and for the Welsh culture and context and is being offered to all state schools in Wales.

**Methods:**

This two-armed cluster randomised control trial (RCT) will evaluate the effectiveness of The Guide Cymru. All 205 secondary schools in Wales will be invited to take part, involving up to 30,000 year 9 pupils. Schools will be randomised to either the immediate implementation of The Guide Cymru or to a wait-list control. The wait-list control will receive The Guide Cymru around 12 weeks later. Measures of mental health literacy (assessed via the Knowledge and Attitudes to Mental Health scale) and mental health problems (via the PedsQL and Strengths and Difficulties Questionnaire) will be taken at baseline (pre-intervention), 12 weeks later (after the active group has received The Guide Cymru), and 24 weeks later (after the wait-list control has received The Guide Cymru).

**Discussion:**

The trial aims to evaluate if The Guide Cymru increases mental health literacy, including reduced stigma to others and to the self, and increased levels of good mental health behaviours and help-seeking for mental health problems.

**Trial registration:**

ISRCTN15462041. Registered 03/10/2019.

## Background

Mental health problems are one of the main causes of the burden of disease worldwide [1]. Mental health services in the UK are currently overstretched, individuals experience long waiting times for treatment, and specialist services are often lacking [2]. Despite this, public spending is focused almost entirely on coping with crisis, with little investment in prevention. Specialist Child and Adolescent Mental Health Services in Wales (CAMHS) is under more pressure than ever before as over the past four years they have observed a 100% increase (from 1204 to 2342 cases) in demand use for the service [3].

Most mental health problems manifest in early life, as one in five children worldwide experience mental health problems [4] with 50% of mental health problems evident by the age of 14 [5]. Only 18–34% of young people with high levels of depression or anxiety seek professional help. In a school-based study of 12 to 17-year-old adolescents, only 18.2% with a diagnosable depressive disorder had ever received support from a mental health service [6].

Poor mental health can have significant consequences, on both general health and on behaviour. For example, it is associated with school drop-out and delinquent behaviours [7]. If unrecognised, or untreated, early-onset mental health disorders may lead to substantial negative personal, social and community consequences. Issues such as these underline the need for an early intervention as part of a comprehensive approach to address mental health needs of adolescents. Early detection of a mental health disorder, coupled with early intervention, has led to better health outcomes and more positive attitudes to mental illness and help-seeking behaviours [8].

The stigma attached to mental health can have significant negative impacts on an individual [9]. Mental health stigma can deter individuals from seeking appropriate mental health support. Barney et al. [10] found that both self-stigma (negative thoughts about oneself) and public stigma (negative thoughts about other people) reduced help-seeking attitudes. Self-stigma can also have significant negative impacts on an individual’s self-esteem and self-worth due to an individual labelling themselves as socially unacceptable [11]. Research has consistently found that individuals protect their self-esteem by not asking for help and by avoiding appropriate help-seeking behaviours [12]. This avoidance mechanism also prevents individuals from seeking help from non-professional sources such as family and friends [13].

In order to promote early intervention among adolescents, interventions need to target stigma reduction as the stigma attached to mental health has been shown to prevent help-seeking behaviours among adolescents [14]. Research suggests that enhancing an individual’s mental health literacy can aid stigma reduction through the improvement of public knowledge of mental health and increasing attitudes towards help-seeking behaviours - particularly among adolescents [15]. Mental health literacy originates from the domain of health literacy, which aims to improve patient knowledge about physical health and treatments in order to promote and improve physical health [16]. It has been proposed that mental health literacy programs may play an important role in facilitating access to care among young people with mental health problems.

Mental health literacy has many components, including (a) prevention through good mental health behaviours, (b) symptom recognition, (c) self-help strategies, (d) help-seeking strategies and treatment options, and (e) how to support others in need [17]. This definition of mental health literacy lies among current theories of health promotion, such as stigma theory, which claims that a lack of knowledge is a driver of prejudice (negative attitudes) which, in turn, influences negative behaviour (discrimination) [18]. This model of ignorance leading to discrimination suggests that it is crucial to improve the mental health literacy of adolescents in order to counteract stigmatic-related beliefs about mental health problems.

Many individuals with mental disorders internalise these negative stereotypes about mental disorders, and this negative self-perception has become known as self-stigma. Self-stigma results in harm to self-esteem, self-efficacy, and quality of life [19]. Research has shown that educational programs targeting self-sigma reduction have resulted in higher self-esteem, better quality of life, and increased social support among individuals [20], and improvements in the mental health literacy of adolescents have been shown to be related to increased resilience and control over their own mental health and increases in self-helping behaviour [21].

Currently in the UK there are low levels of mental health literacy in secondary schools [22]. However, educators, health professionals, and policy makers have recognised the importance of schools in being able to address the mental health needs of young people [23]. Hence, a mental health literacy programme that is delivered to all children as part of the school’s curriculum could produce a reduction in mental health related stigma and an increase in help-seeking behaviours for mental health problems, with the hope that this in turn would improve the mental health of these children in the short-term, and as they develop into adults over the long-term.

Action for Children (AfC), a third sector UK charity, have received funding from the Welsh Government to deliver a mental health literacy programme (The Guide Cymru^1^) to all Year 9 pupils (age 13–14) in main stream education throughout Wales. The Guide Cymru is based on an evidence-based intervention and resource aimed at improving knowledge and attitudes towards mental health in adolescent populations [25]. The Guide (Cymru) compromises of six modules including; 1) understanding mental health and mental illness, 2) stigma myths and realities, 3) information on specific mental illnesses, 4) experiences of mental illness, 5) help-seeking and finding support, and 6) the importance of positive mental health. In Canada, the Guide has led to significant improvements in mental health knowledge and stigma in high school student aged 16–17 year olds [25]. The Guide Cymru is based on The Guide but has been revised to be appropriate for a lower age group (12–15 year olds) in order to target the key age of onset of many mental health problems. It has also had its language and terminology revised to fit the culture of the UK/Wales. A Welsh-language version of The Guide Cymru was also produced.

This randomised control trial (RCT) will evaluate the effectiveness of the mental health literacy intervention within a Welsh culture. The Guide Cymru will be delivered to teachers who will then deliver the content to a cohort of all Year 9 pupils (aged between 13 and 14 years) across Wales. While previous evaluations [25] of The Guide have examined knowledge of mental health and attitudes towards mental health, we aimed to take a more detailed assessment that broke this down into knowledge of mental health, good mental health behaviours, stigma to others, stigma to the self, avoidance coping, and help-seeking behaviours.

We hypothesise that children that receive The Guide Cymru will show greater mental health literacy as indicated by: (1) greater knowledge of mental health issues, 2) better mental health related behaviours, (3) less stigma towards those with mental illness, (4) reduced levels of self-stigma, (5) a reduction in avoidant coping styles, (6) a greater intention to seek help if they have a mental health problem.

## Method/design

**Fig. 1:**
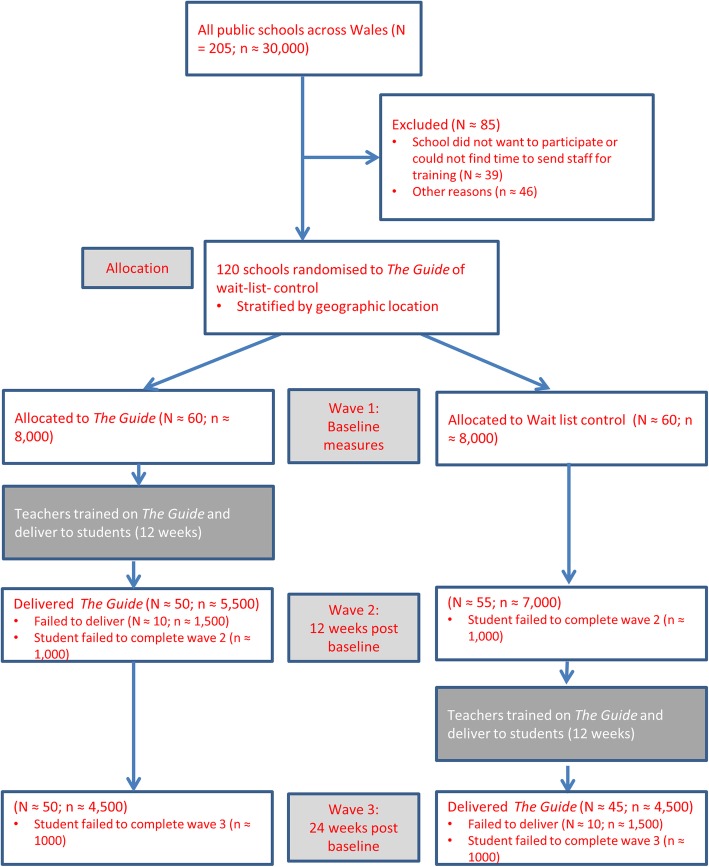
A Consort Representation of Study

A CONSORT statement was used to describe the study [26] – see Fig. 1. The study design is a two-armed cluster-randomised wait list control trial of The Guide Cymru mental health literacy programme. The setting is the Year 9 pupils (age 13–14 years) in state schools in Wales.

A cluster-randomised control trial was deemed necessary as The Guide Cymru training programme is delivered to the students via their teachers and so the school is the unit of clustering. Hence, randomisation will take place at the level of the school and will involve clusters of approximately 150 (range approx. 30–300) students. However, the main outcome (e.g., mental health literacy) will be at the level of the individual students and not at the cluster level.

### Recruitment and consent

All schools in mainstream education in Wales will be sent a letter to their Head-teacher offering free training for up to three teachers on a mental health literacy intervention: The Guide Cymru. This is an evidence-based intervention [25] and resource that can be used as part of the Personal, Social and Health Education curriculum or as part of the Health and Well-being area of Learning and Experience, within each school. Schools that sign-up for the mental health literacy intervention will be stratified by geographical location into groups of around 10 schools. Schools from this group are then randomly allocated to each arm of the trial (The Guide Cymru vs wait list control).

The Guide Cymru is being adopted as part of the National Curriculum and is therefore delivered to all children within the year group at the school and at the teachers’ discretion, without the need for consent from the parents/caregivers. Data collection will be performed in the classroom via pencil and paper questionnaires. Completed questionnaires will contain no personal information to preserve anonymity. However, the participants will be asked four “code” questions that allow for the tracking of the person across the three waves (“name of first pet”, “date of your birth day (e. g,.23^rd^)”, “house number”, and “favourite colour”). Ethical permission for the study has been obtained from Swansea University (Approved 28/10/2018, Department of Psychology Ethics Committee, ref.: 2018–0272-259). Following completion of the Guide Cymru, participating schools will receive general feedback on pupils knowledge, stigma and reported help-seeking behaviours in relation to mental health. No individual pupil data will be reported as only group data will be analysed.

### Randomisation

The randomisation process will take place at the level of schools that wish to be trained on The Guide Cymru (estimated *n* = 120). Schools will be stratified by geographic area. This will help to ensure that the schools in the intervention and wait list control are from similar backgrounds, such as the socio-economic status of the students and their use of the Welsh language, etc.. A computer-generated random sampling procedure will be used to ensure unbiased allocation to each group: (https://www.random.org/). After randomisation, each school allocated to the intervention group will be “yoked” to a school in the wait-list control so that everything that is done to the first school is done to the yoked school in wait list control (e.g., day of testing on each wave of testing). Throughout the study, the randomisation process will be conducted by the acedemic leads of the project, without any influence of the project team.

### Blinding

Schools will be randomised to either the intervention group or the wait list control. After they have signed and returned their Service Level Agreements. Given the nature of the intervention it will be impossible for the schools and students to be blinded as to which arm of the RCT they are in.

### Inclusion/ exclusion criteria

Schools are eligible to participate if:

1. They are a mainstream state school in Wales.
2. They wish to deliver the intervention as part of their curriculum to Year 9 pupils.
3. They are able to send up to three members of staff to a two-day training programme.

Schools are not eligible to take part if:

1. If they are a school outside of Wales
2. If they are a primary school, a special school (for young people with Learning Disability or Behavioural difficulties), or an independent sector school.
3. If they are unable to send at least two teachers to the teacher training programme (see below).
4. They are not to be able to deliver The Guide Cymru intervention following receipt of the training programme.

Young people are eligible if they meet the following criteria:

1. Aged 13–14, in Year 9 of secondary school, attending a mainstream state school in Wales.
2. Understand written and spoken English or Welsh.

Young people are not eligible to take part if:

1. They are not currently being educated in a state school in Wales in year 9.

### Intervention

The Guide Cymru is an intervention and resource aimed at improving knowledge and attitudes towards mental health in adolescent populations. It is based on The Guide [25] which was originally written for and tested on 16–17 year olds in North America. The Guide (and therefore The Guide Cymru) includes 6 modules to mental health including; Understanding Mental Health and Mental Illness, Seeking help and the importance of positive mental health. The Guide has evidenced sustained improvements in student’s mental health literacy in Canada [27].

The Guide Cymru was adapted by Action for Children (a UK based charity) via a team that included psychologists, senior teachers, a child psychiatrist, and children (age 14) to be more appropriate for the intended age group (13–14 year olds) and the Welsh setting. After adaptation, all materials were translated into Welsh for delivery in this language if the school wished.

The Guide Cymru includes a two-day training course for teachers (termed the Go-To Educator) that covers the materials in The Guide Cymru and the learning resources available. Each school that signs up to the intervention programme will be invited to send up to three teachers (free of charge) to this training. These teachers will then deliver The Guide Cymru to their students over a next 8–10 weeks.

### Procedure

Data will be collected in three waves.

#### Wave one

The aim of wave one is to provide baseline measures of mental health literacy, and levels of psychological function and mental health difficulties. The wave will occur approximately 1 week prior to these teachers being trained on Go-To Educator and then delivering the intervention programme to their students over the following 10 weeks. A member of our research team will visit each school on the day of testing to help the teachers administer the questionnaires to the students.

For each school in the intervention arm of the RCT there will be a “yoked” school in the wait list control arm and the data from the yoked school will be collected on the same day and in the same manner as the one in the active arm.

We will determine Social Economic Status (SES) and special needs status of the schools through the Welsh Government Annual Documentation detailing a percentage of many pupils are eligible for free school meals and how many require additional learning support.

#### Wave two

The wave will occur approximately 1–2 weeks post the delivery of The Guide Cymru for the students in the intervention arm of the study, with the wait list control yoked schools being tested at the same time.

#### Wave three

Wave three will occur approximately 1–2 weeks post the delivery of The Guide Cymru for the students in the wait list control arm of the study, with the yoked school in the active arm being tested at the same time (which will be approximately 14 weeks after these schools had completed the mental health literacy intervention).

### Primary outcome measure

#### Knowledge and attitudes to mental health (KAMH, Simkiss, Gray & Snowden, in preparation)

The KAMH measure is a questionnaire designed for children and adolescents aged 11–16. The KAMH instrument measures mental health literacy over 6 domains: (1) Mental health knowledge, (2) Mental health stigma (lack of), (3) Self-stigma (lack of), (4) Good mental health behaviour, (5) Avoidant coping (lack of), and (6) Help-seeking behaviour. It also contains a social desirability scale to measure and control for positive impression management. Participants respond to statements on a five-point Likert scale (strongly agree, agree, don’t know, disagree, strongly disagree) with higher scores indicating higher mental health literacy (e.g. greater knowledge, less stigma, more help-seeking, etc.). The scales of the KAMH have shown good psychometric properties, including good internal reliabilities and test-retest reliabilities (Simkiss et al. in preparation).

### Secondary outcome measures

#### PedsQL

The Pediatric Quality of life inventory (PedsQL) 4.0 Generic Core Scales [28] is a health-related quality of life measure that has demonstrated good reliability and construct validity in various populations [29]. The PedsQL 4.0 Generic Core Scales instrument consists of the following 4 domains: (1) Physical Functioning, (2) Emotional Functioning, (3) Social Functioning, and (4) School Functioning. It includes format for typically developing children aged 5 to 18 years old. Participants rate each description regarding to their heath over the past month using a five point Likert scale (never, almost never, sometimes, often, almost always). Item scores that then transformed to a score out of 100 with high scores indicating a high quality of life.

#### Strengths and difficulties questionnaire

The Strengths and Difficulties Questionnaire (SDQ) [30] assesses the psychological adjustment of children and youths. The SDQ has 25 items, both positive and negative, answered using a 3-point Likert scale (not true, somewhat true, certainly true) to indicate how far each item applies to the individual over the past 6 months. The 25 items are divided between five scales of five items each, generating scores for emotional symptoms, conduct problems, hyperactivity-inattention, peer problems and prosocial behaviour. Emotional, conduct, hyperactivity- inattention and peer problems are summed to generate a total difficulties score. Higher scores indicate more difficulties. The SDQ has well documented psychometric properties [30].

### Statistical analysis

#### Descriptive statistics

Descriptive statistics for the samples (e.g., age, gender, socioeconomic status) will be provided. Mean scores (with standard deviations) will be calculated and presented for each of the three waves of evaluations.

#### Primary analysis

Multi-level modelling (MLM) will be used to test all hypotheses. Our primary analysis will compare the measures at wave 1 and wave 2. We expect to see time by group interactions, such that there are improvements in mental health literacy (e.g., knowledge score on KAMHs) from wave 1 to wave 2 for the treatment group but not for the wait list control.

#### Secondary analyses

We will examine the data from wave 3 to see if the treatment group has maintained its hypothesised increase in mental health literacy. We will examine if the wait list control (who will now have been administered The Guide Cymru) show a similar increase in mental health literacy and quality of life from their performance at wave 2.

We will examine factors that might moderate the effectiveness of The Guide Cymru, such as gender, Social Economic Status (SES) of the school, etc. by adding these into the MLM as a fixed factor and examining any significant interactions with these factors.

Pilot experiments have shown the data from the KAMH scales have an approximately normal distribution. Data from the PedsQL and SDQ are often positively skewed so medians and inter-quartile ranges will be presented, and the data will be transformed for the inferential statistical analyses.

#### Statistical power

A power analysis was conducted to determine appropriate sample size. Our cluster size is determined by the number of secondary schools across Wales with there being approximately 205 schools in mainstream education with approximately 30,000 pupils in year 9 or around 150 pupils per school (though this cluster size will vary greatly across schools).

A normal (non-clustered) RCT power analysis with parameters of alpha = .05, power of 80%, and a standardised effect size of 0.30 (a small effect size for Cohen’s *d* and close to that obtained by [25] requires 175 pupils per group (*N* = 350). To account for the reduction in power due to clustering we assumed an average cluster size of 150, and an intra-cluster correlation coefficient (ICC) of 0.10, which leads to a design effect of 16 [25]. Hence, we require a sample size of around 2800 per group (*N* = 5600). Our predicted final sample size of 13,000 exceeds this (see Fig. 1) and allows a margin of error if our estimates of take-up or intra-cluster correlations are inaccurate.

### Data management

Completed questionnaires will contain no personal information to preserve anonymity. Data entry will be completed electronically. A minimum of 10% Veracity checks will be completed on all data entry. All databases will be secured with password-protected access systems. All participant questionnaires will be stored in locked file cabinets in areas with limited access.

## Discussion

This study will evaluate a mental health literacy programme, The Guide Cymru, in a large sample of year 9 (age 13–14 students) across a whole country (Wales, UK). The Guide Cymru is adapted from The Guide [25] which has shown to be effective in increasing mental health literacy in older children (age 16–17) in a North American sample. This move to a lower age group was felt important given the early onset of many mental health problems and that 50% of 14 year old girls are estimated to experience mental health difficulties [5]. However, the earlier finding that The Guide was not effective for students on a lower academic stream [25] suggests its downward extension to a lower age group may limit its effectiveness.

### Strengths of the study

A major strength of the study is that the Guide Cymru will be tested in situ with the teachers who are able to adapt the course and materials to the needs of their own classroom and school. Hence, this provides a much clearer picture of the expected benefits of The Guide Cymru than might be provided by the intervention programme being delivered by “experts” on this topic.

A second strength is the large-scale nature of the study with over 200 schools and therefore up to 30,000 students being trained on the programme and evaluated for outcome. While not all schools will be able to take part (see below) a large sample size will allow for a more fine-grain analysis of which schools were able to benefit from the intervention.

Finally, the possible benefits of The Guide Cymru will be assessed in more detail than previous research by the use of a questionnaire (KAMH) that examines mental health literacy along several dimensions: Knowledge, stigma towards those with mental health problems, self-stigma, good mental health behaviours, avoidant coping, and help-seeking. This analysis will be able to evaluate what aspects of mental health literacy are improved, or not improved, by The Guide Cymru and therefore provide important information for how the intervention might be developed further for maximal impact in our young people. We will also take measures of current mental health to examine the relationship between mental health literacy, attitudes, and mental health.

### Challenges and limitations

A major challenge is to be able to deliver the intervention within the time period of the funding. The intervention is delivered over a 8–10 week period (typically) by the schools themselves after the teachers have been trained in the delivery. Hence, the intervention has to be integrated into the curriculum of the schools which are often planned months, if not a complete year, in advance. Hence, while schools might be keen to take advantage of this free resource (funded by Welsh Government), the practical difficulties in assigning teachers to be trained (including inevitable drop-outs, transfers, sickness, etc) and the integration of the intervention programme into the school timetable will limit the number of schools that will be able to deliver the Guide Cymru.

The second limitation is that we will not be able to identify individuals due to the anonymous nature of the data collection. Hence, we will not be able to look at factors such as academic achievement or socioeconomic status as mediating factors in the success/failure of the intervention other than at the level of the school.

Finally, while we will follow students over three phases of data collection that span approximately 6 months. This follow-up period is short compared to the hoped-for benefits of the intervention. We hope that if we are able to show significant benefits within this short-term follow-up then this will encourage further development of mental health literacy programmes along the lines of The Guide and The Guide Cymru. These mental health intertervention programmes within our schools can then become part of the continuing education of students across several years of study, so as to reinforce and consolidate this learning and help young people to develop positive attitudes towards mental health difficulties and help-seeking at times of need, alongside behaviours that promote good mental health.

## Data Availability

The datasets generated during and/or analysed following the current study will be stored in a publically available repository such as Mendeley. The data shared will be an anonymised SPSS database that contains the item by item scores from the questionnaires as well as the scale scores and demographic information. The data will be published at the time of submission of the paper describing the results of the RCT and will remain available indefinitely. Access will be open to anyone via the usual access to Mendeley.

## Abbreviations

AFC: Action for children
CAMHS: Child and adolescent mental health service
ESF: European social funds
KAMH: Knowledge and attitudes to mental health
KESS: Knowledge economy skills scholarship
MHL: Mental health literacy
MLM: Multi-level modelling
PedsQL: Pediatric quality of life inventory
RCT: Randomised control trial
SDQ: Strengths and difficulties questionnaire
SES: Social economic status
SPSS: Statistical package for the social sciences
UK: United Kingdom

## Footnotes

1 Cymru is the word in the Welsh language for “Wales”.
